# Repetitive stability study of remdesivir/cyclodextrin complex on the international space station

**DOI:** 10.1038/s41598-024-81428-5

**Published:** 2025-02-04

**Authors:** György Dormán, Balázs Buchholcz, István Puskás, Pál Szabó, Erzsébet Varga, Lajos Szente, György M. Keserű, Ferenc Darvas

**Affiliations:** 1 Innostudio, Inc., Záhony u. 7., Budapest, 1031 Hungary; 2 Institute of Chemistry, Budapest University of Technology and Economics, Műegyetem rkp. 3., Budapest, 1111 Hungary; 3 Cyclolab Cyclodextrin Research & Development Laboratory Ltd, Illatos út 7., Budapest, 1097 Hungary; 4 Research Centre for Natural Science and National Drug Research and Development Laboratory, Magyar Tudósok körútja 2., Budapest, 1117 Hungary

**Keywords:** Remdesivir sulfobutylether-beta-cyclodextrin, In-space drug stability, Space medicinal chemistry, Space environment, Chemical degradation, Reproducibility, Standardization, Drug discovery, Astronomy and planetary science, Chemistry

## Abstract

Stability assessment of drugs in space is particularly important for future missions. In space there are multiple factors, such as the variability of the conditions (radiation, microgravity, vacuum etc.) that could affect the reliability and reproducibility of the data. Therefore, we investigated the stability of an anti-Covid drug formulation, Remdesivir (RDV) sulfobutylether-beta-cyclodextrin (SBECD) complex, in two separate flight experiments on the International Space Station (ISS). While HPLC/MS studies revealed no degradation of the cyclodextrin excipient in any of the samples investigated in both missions, RDV purity analysis of the RDV/SBECD complex after the first mission revealed different stabilities and altered degradation in space and on Earth. This latter interesting finding was not supported by the second mission, where no differences in the drug stabilities were identified. This anomaly highlighted the importance of standardization together with increased control of the variable parameters during the entire space missions and the terrestrial control experiments.

## Introduction

Chemical stability of drugs and bioactive agents is a critical issue concerning long-term space missions and has led to the emergence of space pharmaceutical chemistry^1,2^. It is obvious that the stability of drugs used for short- or long-term space missions cannot be evaluated by drug stability guidelines based on terrestrial environmental factors^3–5^ and requires different and space-validated approaches. Numerous space characteristics, including radiation, vacuum, magnetic field, spaceship transition conditions (excessive vibration, fluctuation of gravity), temperature changes, and humidity variation, could all influence the chemical stability of the drug components. Different forms of radiation (nonionizing radiation, ionizing radiation, solar flares and particles trapped by the Earth’s magnetic field) are considered the most important factors affecting the stability of medicines. Furthermore, microgravity in space could lead to disintegration of certain formulation such as tablets.

At the same time microgravity could offer largely unexplored opportunities producing altered or improved crystal morphology, different structures of microparticles (e.g., polymorphism), and several examples demonstrate beneficial effects on the formulation^6–10^ as well as on the chemical stability^11^. Under microgravity, diffusion is the only driving force since the lack of the preferred direction of the mass transfer makes the convection negligible^12^.

Certain excipients, such as cyclodextrins^13^, that are frequently used to improve the stability^14^ and solubility^15^ of active pharmaceutical ingredients (APIs) through forming inclusion complexes. Interestingly, cyclodextrins show radiation shielding effects that could prevent oxidative damage^16^. Based on the above considerations, we have chosen an API formulation with extremely high cyclodextrin content. Remdesivir is a prototypical API that has serious solubility^17^ and stability^18^ problems. Although intravenous administration enables its full absorption, hydrolysis-mediated first-pass clearance makes oral use impossible due to limited absorption and low systemic exposure^19^. The low aqueous solubility at neutral pH or slightly acidic media represents a serious drawback even of parenteral administration. The dissolvable powder formulation remdesivir (RDV) therefore contains 97% sulfobutylether-beta-cyclodextrin (SBECD) to solubilize the API and the inclusion complex might have effect on stability that was investigated in two separate experiments on the International Space Station (ISS).

Such space experiments could provide important observations regarding the chemical stability changes of the components, degradation mechanisms, as well as changes in the crystal structure and formulation. We were aware that many challenges and unsolved issues could seriously influence the outcome of the actual space experiments, leading to uncertain, inconsistent results due to numerous presently unknown and/or uncontrolled variabilities. Thus, it could raise the importance of repeating the experiments ensuring identical circumstances as much as possible to reproduce and confirm the results obtained. Fortunately, we had a unique opportunity to repeat the experiment in two separate spaceflights on the International Space Station (ISS), including the sample generation and delivery, time and duration of the spaceflight. After returning the RDV content of the complex, the chemical stability of RDV and SBECD, and the degradation profile of the drug substance were investigated in both cases. Here we describe the key steps of the entire space experiment and the applied methods and discuss the results of the study attempting to rationalize the unexpected outcome of the experiments. Finally, we summarize the key challenges of such space experiments with suggestions for future missions.

### Investigation of the remdesivir–cyclodextrin complex at the ISS

Innostudio, Inc. together with its project partners (Cyclolab^20^, Hungary and JAMMS, Japan^21^) were the pioneers in testing a drug dissolvable powder formulation containing cyclodextrin carrier in the Space. Between December 2020 and February 2022, the team performed two experiments at the ISS to investigate the remdesivir formulation (the third experiment is ongoing). In collaboration with Japan Manned Space Systems Corporation (JAMSS), two experiments were conducted at the ISS. In the first experiments, two different samples were investigated in space and on Earth: one with neutral and one with acidic conditions. During the second mission, two identical samples were investigated at neutral pH both in space and on Earth.

Delivery of the samples to the ISS and carrying out the experiment was done in the Columbus module in collaboration with JAMSS, Space Applications Services (SAS)^22^, and the European Space Agency (ESA)^23^. The samples that were retained on Earth and travelled into space were subjected to extensive analysis, including HPLC‒MS and MS/MS studies, to identify API degradation, its patterns and degradation products for both the terrestrial and space-based samples.

The samples returned from space together with the terrestrial samples were investigated for RDV content, purity and RDV/SBECD chemical degradation (in the liquid fractions of the suspensions). The terrestrial samples followed the same continental route except they stayed on Earth, while their counterparts spent ca. 30 days on the ISS).

## Materials and methods

Sulfobutylether beta cyclodextrin (SBECD) was a product of Cyclolab Cyclodextrin Research & Development Laboratory Ltd. (Dexolve®), Hungary, batch 47K330720 (complying with United States Pharmacopoeia 43). Remdesivir was a product of Echemi, China batch L-185/20 (purity: 99.6% by HPLC), and the nominal RDV/SBECD weight ratio was 9.1% in the test suspension samples.

The experiments were carried out in the JAMSS’s KIRARA cubes^24^ that were originally developed for protein crystallization. We used a vial containing a crystallization capillary as a vessel for our experiments. The ICE Cube end-to-end service platform^25^, which hosts the KIRARA cubes, maintains and operates the formulation process provided by the SAS^24^ on the ISS. The ICE Cube facility allows 100 individual experiments in a 10 cm × 10 cm × 10 cm cube (so-called 1 U (unit)). The platform itself is housed in the ISS Columbus Laboratory^26^.

### Logistics and transportation

The journey started with lab experiments via sample preparation at the laboratories of Cyclolab in Budapest, Hungary, with the participation of Innostudio, Inc. Two identical sets of samples were prepared. The samples were delivered to Tokyo to the laboratory of Confocal Science Inc.^27^, where they inserted the samples into two identical experimental vessels: a space sample and a control (ground) sample. The vessels were packed into the KIRARA sampler holder cube, which is compatible with SAS’s ICE Cubes platform (ISS device). KIRARA Cube is a device for commercial space applications, maintained by the Japan Manned Space Systems Corporation. The main function of the device is crystallization; however, the crystallization vessels can also accommodate other types of chemical experiments. The cube has dimensions of 10 × 10 × 10 cm (1 unit). The device can store dozens of samples. Each sample is carefully sealed in gel tubes without headspace, which are enclosed in sealable bags.

**Fig. 1:**
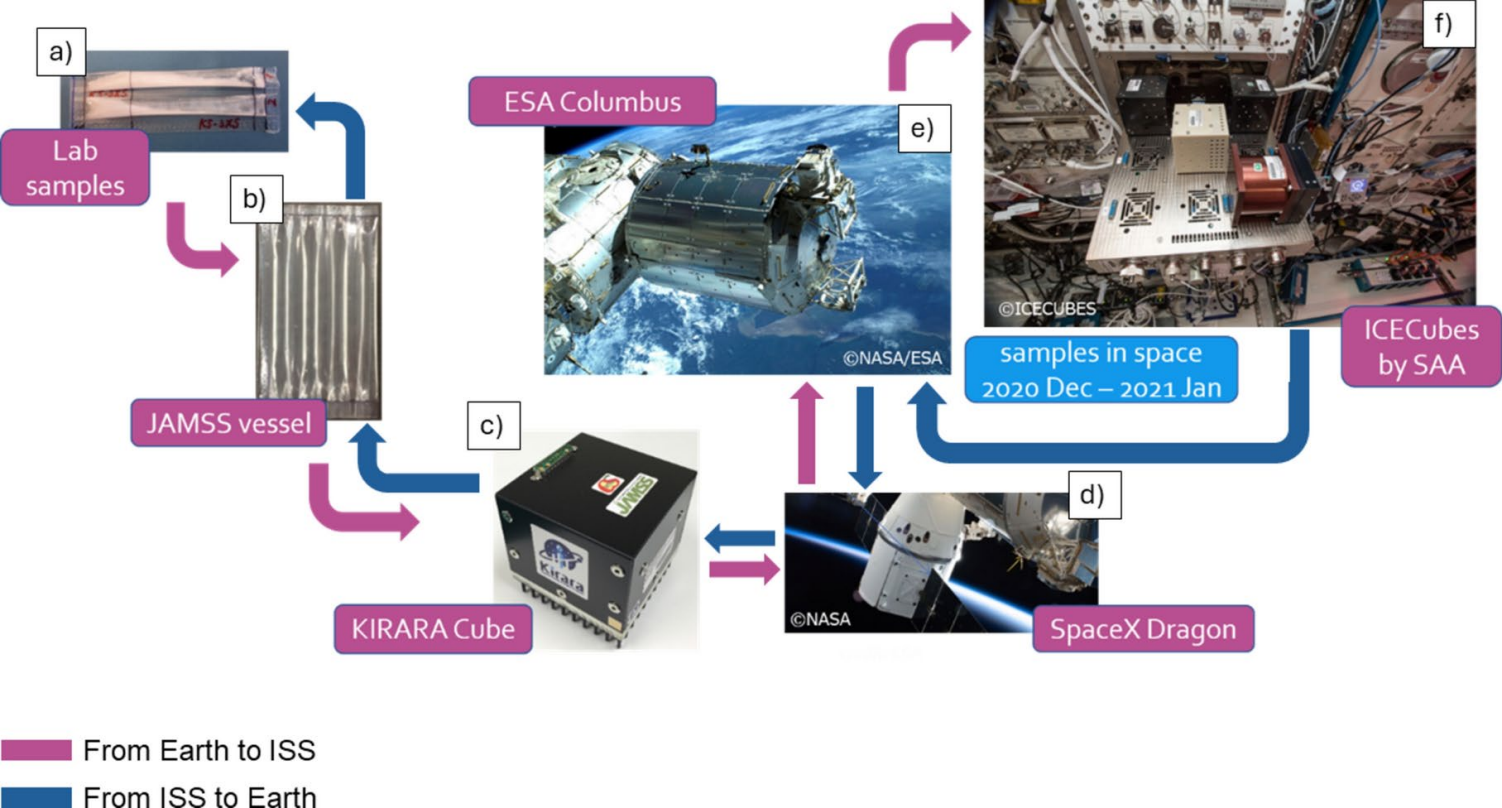
The journey of the samples from Earth to space and back. Samples prepared on Earth in the laboratory of Cyclolab Ltd. (**a**) were sent to the laboratory of Confocal Science Inc. in Japan for preparation (**b**) and were placed in the KIRARA cube of the Japan Manned Space Systems Corporation (**c**). The KIRARA cube was transported to the ISS Columbus laboratory (**e**) aboard the SpaceX Dragon spacecraft (**d**) and was placed on the Space Application Services ICE Cubes platform (**f**). The samples returned to Earth following the same route. The outward journey is indicated by magenta lines, while the return journey is indicated by blue lines.

The KIRARA cube with the vessels inserted was delivered by a flight to the launch station (Kennedy Space Center) in Florida, where it was packed to the SpaceX-21 rocket and sent to the ICE Cubes facility of the ESA’s laboratory on the ISS (Fig. 1). The samples stayed on the ISS for 31 days. After finishing the experiments, the same route was followed to recollect the samples for processing at Cyclolab and Innostudio. The control samples were kept on Earth at the Tokyo site of JAMSS and were forwarded to Innostudio in Hungary together with the space samples. (Mission #1: 2020 Dec.—2021 Jan.; Mission # 2: 2021 Dec.—2022 Jan.).

### Sample preparation

#### Sample preparation before the space missions

##### Neutral sample (1st and 2nd mission)

A total of 400 mg (0.185 mmol) of solid SBECD (on dry basis) and 40 mg remdesivir (0.066 mmol) were homogenized thoroughly for 5 min in an agate mortar with a pestle and then quantitatively transferred into a glass vial of high hydrolytic resistance (Type I) of 5 mL volume. The powders were suspended in 1.6 mL of Millipore Synergy® ultrapure water by vortexing (500 rpm, 1 min) at room temperature. The hydrophilic SBECD was fully dissolved, but remdesivir was only partially solubilized: the resultant liquid was a white suspension. The pH of the suspension medium was 6.5.

A 1.1 g representative sample was sent to the laboratory of Confocal Science Inc. in a 5 mL hydrolytic class I vial stoppered by bromobutyl elastomer stopper and aluminum cap.

##### Acidic sample (1st mission)

A total of 400 mg (0.185 mmol) of solid SBECD (on dry basis) and 40 mg remdesivir (0.066 mmol) were homogenized thoroughly for 5 min in an agate mortar with a pestle and then quantitatively transferred into a glass vial of high hydrolytic resistance (Type I) of 5 mL volume. The powders were suspended in 1.6 mL diluted hydrochloric acid of pH 1.9 prepared with Millipore Synergy® ultrapure water by vortexing (500 rpm, 1 min) at room temperature. The hydrophilic SBECD was fully dissolved, but remdesivir was only partially solubilized: the resultant liquid was a white suspension. The pH of the suspension medium was 3.5.

A 1.1 g of the corresponding samples were sent to the laboratory of Confocal Science Inc. in a 5 mL hydrolytic class I vial stoppered by bromobutyl elastomer stopper and aluminum cap.

For KIRARA, each sample was separated into two equal parts by micropipette. Separated slurries were transferred into Sealbag Gel Tubes (SGT). SGT is a quadruple, elongated cylindrical cell made of a PET sheet with high gas barrier properties. SGEs were placed in the KIRARA cube, while the Peltier module kept the temperature of the samples at 20 ± 2 °C. Temperature was monitored from sample installation until unboxing.

#### Sample preparation for analytical measurements after returning

The samples were processed for investigations after return to Earth. The control terrestrial samples were processed in an identical manner. The samples were transferred to 1.5 ml Eppendorf tubes. The supernatant and the solid particulates were separated by centrifugation (Hermle Z 216 MK, 25 °C, 20 min, at 13,300 m s^−2^ relative centrifugal force). After separation, the supernatant was separated by an automatic pipette, and the solid sediment was washed by the following procedure step-by step:

- Addition of Millipore Synergy® ultrapure water in volume equivalent to that of the removed supernatant
- Vortexing (500 RPM speed, for 20 s)
- Centrifugation
- Removal of the supernatant

As a result, four supernatant samples were obtained:

- G1: terrestrial/control sample (neutral sample)
- S1: space sample (neutral sample)
- G2: terrestrial/control sample (acidic sample)
- S2: space sample (acidic sample)

### HPLC methods

Three HPLC methods (A, B and C) were designed and used for different purposes and answering different questions.

*Method A* was designed to investigate the cyclodextrin component (SBECD) and its possible inhomogeneities, chemical degradation products, as supported by the quality of the stationary phase (ion exchange column) and detection method (ELSD).

The second method (*Method B*) was developed for the quantitative analysis of API content (remdesivir) and its degradation products in line with methods previously presented in the literature. Here the aim was to determine the distribution of API and its impurities and to verify reproducibility for API measurements. On this basis, quantitative conclusions could be drawn on the amount of the components (API, degradation products) in the ground and ISS samples. This was achieved by separation of API and degradation products in a classical reverse phase chromatography system. Quantitation was based on absorbance measured at UV 240 nm.

*Method C* provided the qualitative information and was mainly used for the analysis of degradation products and degradation pathways. *Methods B* and *C* use similar stationary phase, mobile phase (eluent composition, flow rate) and the aim is to determine the structure of the degradation products present in *Method B*.

#### HPLC method for SBECD chemical degradation analysis (fingerprint investigation, Method A.)

A total of 1.25 mL of the supernatant was diluted in 5.0 mL of Millipore Synergy® ultrapure water. The samples were analyzed by an Agilent 1260 HPLC Instrument equipped with a CD-Screen-IEC column (3 μm, 150 mm × 4.0 mm 4-dimethylamino benzyl carbamide silyl silica gel for chromatography stationary phase from Bio-Sol-Dex, Hungary).

The following eluents were used:

*Mobile phase A*: Chromatographic-grade water (900 mL) was added to 4.0 mL of triethylamine, adjusted to pH 4.5 with anhydrous formic acid, and then diluted to 1000 mL with water for chromatography. *Mobile phase B*: acetonitrile.

The following instrumental parameters were used for the analysis: gradient: 0–14 min, B% = 10–60; flow, 1.1 mL/min; injection volume, 10 μL; and detector, evaporative light-scattering detector. For the Agilent ELS detector, the carrier gas was nitrogen; the flow rate, 1.2 L/min; the evaporator temperature, 50 °C; and the nebulizer temperature, 30 °C. The detector parameters for Sedex-100 ELSD were as follows: carrier gas, nitrogen; gas pressure, 3.2 ± 0.7 bar; and temperature, 70 °C.

#### HPLC method for RDV purity and chemical degradation analysis (method B)

HPLC analysis was conducted to determine the RDV content of the supernatant (liquid) fractions together and their purity. An Agilent 1260 HPLC system equipped with a diode-array detector (DAD) was used to identify and quantitate REM. Reversed-phase separation was achieved on a Kinetex C18 analytical column (Phenomenex Inc., Torrance, CA, USA) with 100 mm length, 4.6 mm internal diameter and 2.6 µm particle size using a solvent gradient of water with 0.05% formic acid with acetonitrile at 0.05% (0 min 0%, 10 min 100%) at a flow rate of 0.8 mL/min and UV-diode array (DAD) detection at 240 nm wavelength.

For confirming the reliability of the HPLC measurements we carried out reproducibility investigations. We measured 2 replicates on 5 different days and each sample twice. In all cases the standard deviations were less than 10%, which corresponds to the max 15% standard deviation required for pharmaceutical applications. Reproducibility of the RDV HPLC UV/MS measurements is shown in Table S1.

#### HPLC‒MS method for RDV chemical degradation analysis (method C)

The supernatant (liquid) fractions were subjected to HPLC‒MS degradation studies. The measurements were carried out on a Sciex 5600 + Triple TOF high-resolution mass spectrometer equipped with a DuoSpray ion source operated in positive electrospray mode. The mass spectrometer was scanned in TOF–MS mode in the mass range of 100–1000 Da, and the accumulation time was 1 s. The resolution was above 25,000 over the entire mass range. A Shimadzu Prominence LC20 system was coupled to the mass spectrometer. The LC system consisted of a binary pump, an autosampler, a column oven and a UV/VIS detector. A Kinetex C18 column (100 mm × 3 mm, 5 µm) was used for the separation of the samples. HPLC-grade water (eluent A) and acetonitrile (eluent B), both containing 0.1% formic acid, were used as mobile phases. The flow rate was 0.8 mL/min. Two microliters of sample was injected. The column was kept at room temperature. A 240 nm wavelength was used in UV detection. Compounds were identified on the basis of retention times and the elemental compositions obtained from high-resolution data. The accepted mass accuracy was better than 5 ppm. The concentrations of the samples were approximately 1 and 5 mg/mL for the 1st and 2nd mission series, respectively. Based on preliminary measurements, a 20-fold dilution was found to be optimal for the samples. Fifty microlitres of sample was diluted with 950 µL of acetonitrile/water 50/50% containing 0.1% formic acid.

## Results and discussion

### Remdesivir content of the liquid (supernatant) samples

The primary objectives of the RDV space mission were to investigate the API content and its purity within the samples. Here, again, the supernatant liquid portion was analyzed. The original purity of the RDV applied in the studies was 99.29%, as determined by the standard quality assurance protocol^28^. In the acidic sample (1st mission), only small changes were observed, and purity values were basically within the error limit. On the other hand, to our surprise, there was a significant difference between the neutral samples, and the RDV purity in space was almost 6% higher than that of the corresponding Earth sample (Table 1). This unexpected result suggested that space conditions somehow contributed to the stabilization of RDV in the inclusion complex, while the parallel sample that remained on Earth suffered more intensive degradation. Two conclusions have been drawn: (1) non-overlapping analytical results suggested a thorough analysis and comparison of the degradation products and pattern in both neutral cases, and (2) a second space mission is necessary to confirm the above unexpected result.

**Table 1:** RDV purity in the supernatant liquid phase (1st mission).

Sample code	pH	Component	Retention time	Area%
G1 (Earth)	7.0	RDV	7.556	89.07
S1 (Space)	7.0	RDV	7.560	94.66
G2 (Earth)	3.5	RDV	7.542	91.25
S2 (Space)	3.5	RDV	7.545	91.52

Since the most interesting and unexpected result was the supposed stability increase of the neutral sample in space, we planned two parallel experiments with only neutral samples in the second mission see Table 2. where the 3rd and 4th samples were repetition of the 1st and 2nd samples.

**Table 2:** RDV purity in the supernatant liquid phase (2nd mission). Second mission only neutral samples G1′/S2′and its parallel G1′/S2′.

Sample code	pH	Component	Retention time	Area%
G1′ (Earth)	7.0	RDV	7.614	96.47
S1′ (Space)	7.0	RDV	7.613	96.42
G2′ (Earth)	7.0	RDV	7.615	96.86
S2′ (Space)	7.0	RDV	7.614	97.33

Interestingly, the results did not confirm the outcome of the first mission, and all the RDV purity data were nearly identical in the range of 96.5–97.3%, which did not reveal any significant difference in RDV stability (Table 2).

### SBECD degradation

The solubility enhancer cyclodextrin for remdesivir, SBECD (Dexolve®), is a multicomponent isomeric mixture of negatively charged cyclodextrin derivatives in the form of sodium salts. The distribution of the sulfobutyl groups on the primary and secondary sides of the cyclodextrin torus is random. The used SBECD batch had an average degree of substitution of 6.5 sulfobutylether functional groups (SBE = 6.5). For quality control (i.e., according to the United States Pharmacopoeia monograph for SBECD^29^) capillary electrophoresis method is used for the fingerprint characterization of the multicomponent excipient in the samples. As an alternative, superior HPLC method (Method A) was also applied in the present study, which enabled the concurrent, sensitive detection of SBECD degradation products and related substances in addition to separating the randomly substituted anionic cyclodextrin components according to the various degrees of substitution. Method A allowed the detection of impurities coming from cyclodextrin synthesis or that emerged as degradation products, including 4-hydroxybutane-1 sulfonic acid (HBSA) and bis(4-sulfobutyl)ether disodium (DBSA), as well as sulfobutylated linear maltodextrins as potential hydrolytic degradation products^30^.

After the two missions, the RDV/SBECD ratio of the supernatant fraction was determined by applying the standard quality control protocol^31^. It was revealed that in the first mission the equilibrium RDV/SBECD weight ratio remained the same within the experimental error (0.50 ± 0.05% for S1 and G1 and 2.8 ± 0.1% for S2 and G2 samples) in the saturated samples at room temperature. Thus, further investigations predominantly focused on the degradation of the components, which has exceptional importance in long-term space missions.

SBECD degradation of the terrestrial and space RDV/SBECD samples was investigated using Method A for both the neutral (G1, S1) and acidic samples (G2, S2). The analysis revealed that there was no detectable change in the SBECD fingerprint chromatogram in any of the samples, confirming that no degradation or change in the substitution pattern occurred. There was no change in the SBECD fingerprint chromatogram (HPLC ELS) in any of the samples (G1/S1—Fig. S1 and G2/S2—Fig. S2) tested (neither neutral nor acidic); thus, no SBECD degradation product was detected. To the best of our knowledge, this was the first comparative stability study on a cyclodextrin derivative with samples spent a longer period of time in space vs. those remaining on Earth.

This result is particularly important since the quantity of the SBECD component is predominant in the solid formulation (96.77%), while the RDV content is 3.23% in the marketed Veklury®^32^, confirming that SBECD is highly resistant to radiation damage and that microgravity (i.e. an object in space is subjected to acceleration equivalent to one-millionth (10^−6^) of the force of gravity at Earth’s surface which is ∼9.8 ms^−^) and the harsh conditions during rocket launch had no effect on its quality. This result is certainly encouraging to apply this cyclodextrin for formulation improvement, including solubility enhancement and radiation protection in space pharmacies. It has to be noted that SBECD is referred with versatile nomenclature both in scientific discussions and also in pharmaceutical regulatory documents. Handbook of Pharmaceutical Excipients, 9th Edition^32^ lists 18 non-proprietary Names and Synonyms for this single excipient including “Sulfobutylether-beta-cyclodextrin” as used consistently herein and also “Betadex sulfobutyl ether sodium” as termed e.g. in Veklury’s summary of product characteristics.

### Remdesivir degradation

Several HPLC methods were developed for RDV degradation analysis after the discovery of the active substance. The pioneering work focused on detecting the bioactive metabolite responsible for antiviral activity^33^. Dadinaboyina et al.^18^ reported an HPLC‒MS stress degradation study on RDV, which was performed according to the ICH guidelines. RDV was found to be labile towards acidic, basic and neutral hydrolytic and oxidative stress conditions, while it remained stable under photolytic and thermal stress conditions. Altogether, nine degradation products were identified that formed under different (acidic, basic, neutral and oxidative) stress conditions. (Fig. S7A–C, numbering DP1-9.)

Preliminary HPLC analysis using standard quality control methods revealed differences in the G1 and S1 neutral samples obtained from the first mission. (Supernatant samples: S1 94% RDV, G1 89% RDV). For detailed evaluation of such differences, LC‒MS and MS/MS studies were performed to identify any differences in the degradation profile and pathways.

### HPLC‒MS degradation analysis of the G1/S1 neutral samples delivered from the 1st mission

**Fig. 2:**
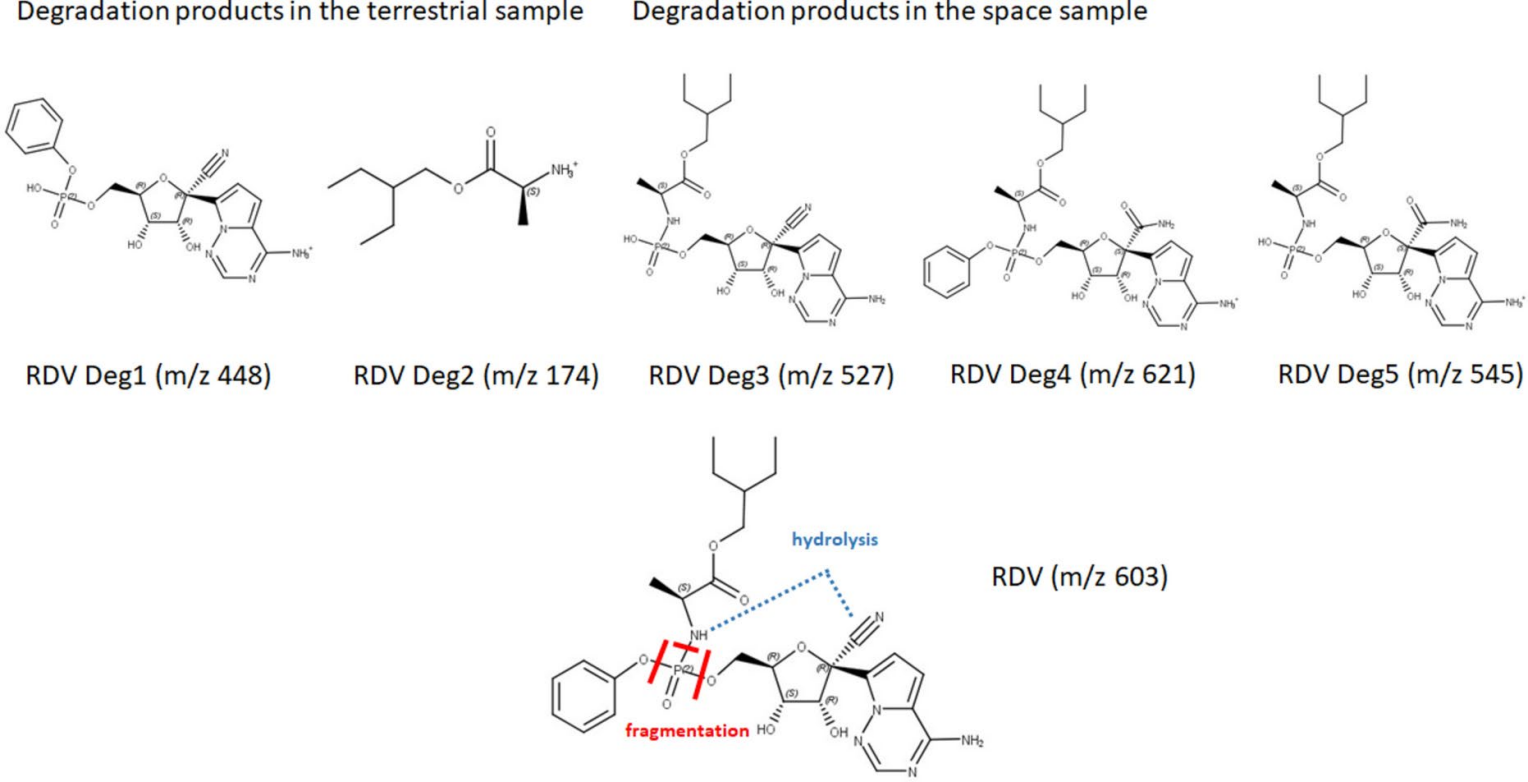
Major degradation products of the neutral samples (1st mission) and their ratio found in terrestrial and in space samples indicating the major reactivity and fragmentation sites of RDV based on Dadinaboyina et al.^18^.

RDV has a retention time (Rt) of 5.86 min (see Suppl. Mat. Figs. S3–S5). Most degradation products elute before this Rt. Peaks eluting after RDV are non-RDV-specific compounds present in the acetonitrile control as well. Based on the high-resolution MS and MS/MS data and with the help of the literature^34^, most of the RDV-related peaks could be assigned. The overlaid extracted ion chromatograms (XICs) helped in comparing the relative amounts of the compounds present in the sample. (see Suppl. Mat. Figs. S4 and S5). The tendency of the differences in the intensities did not give consequent results: an increase and decrease can also be found in the S1 sample compared to the G1 sample. Figure 2. summarizes the main degradation products of RDV identified in the neutral samples of the first mission.

A minor peak appeared at an Rt of 2.66 min with m/z 448.1015 Da (RDV Deg1—Fig. S8, corresponding to DP3^18^—see Fig. S7A). This peak is more intense in the terrestrial sample. The counter pair of this ion elutes at Rt 3.61 min (RDV Deg2—Fig. S9, corresponding to DP4^18^—see Fig. S7A). This is also a minor degradation product with m/z 174.1486 Da. Consistently, its intensity is similar to that of RDV Deg1: more intense in the terrestrial sample. Interestingly, both RDV Deg1 and Deg2 arose from acidic treatment in the terrestrial stress stability study,

There is a characteristic peak at Rt 4.17 min appearing only in the space sample (RDV Deg3—Fig. S10, corresponding to DP8^18^—see Fig. S7B). Another rather intense degradation product appears at Rt 4.6 min with m/z 621.2413 Da (RDV Deg4—Fig. S11, corresponding to DP5^18^—see Fig. S7A–C). The mass difference of this peak and RDV is 18 Da, which is an addition of water supported by the gain in molecular weight at MS peak. This may result from the hydrolysis of the CN group. The intensity of this ion is higher in the space sample. In comparison with the terrestrial stress stability study, these degradation and hydrolysis products appeared solely in basic or neutral media. A similar hydrolysis product of RDV Deg3 was found at Rt 3.4 min with m/z 545.2107 Da (RDV Deg5). This assumption was supported by elemental compositions. It is present only in the space sample, similar to RDV Deg3 and RDV Deg4. It must be highlighted, that RDV Deg5 was not reported in the terrestrial stress stability study.

Based on these results, it can be concluded that different decomposition pathways were found in terrestrial and in space samples. Interestingly, the degradation pathways on Earth corresponded to the acidic treatment in the stress stability studies, while the space-driven routes were similar to those occurring after basic or neutral treatment. Fortunately, neither sample contained oxidative degradation products (e.g., N-oxides), which suggests that SBECD may facilitate sufficient protection from such damage, particularly under increased radiation exposure in space. Finally, all the major reactivity and fragmentation sites together with the feasible pathways corresponded to the outcome of the stress stability studies described by Dadinaboyina et al*.*^18^

The acidic (pH = 3.5) samples (S2/G2) in the first mission also showed comparable stabilities (S2 and G2: 91.2–91.5% RDV). HPLC‒MS analysis revealed that RDV Deg1 (2.644 min, m/z 448.100) and Deg2 (3.54 min, m/z 174.184) were the major degradation products, which corresponds to the “acidic” degradation pathway, not surprisingly, at pH = 3.5.

In summary, RDV degradation analysis confirmed that the S1 neutral samples exhibited higher purity and stability in space than the terrestrial samples (G1) in the first mission, and furthermore, different decomposition pathways were also found on Earth and in space.

### HPLC‒MS analysis of G1′/S1′ and G2′/S2′ neutral samples derived from the 2nd mission

After the unexpected stability outcome of the neutral samples, we attempted to reproduce such results in the 2nd space mission. The four samples of this mission were two parallel pairs: S1 and S2 and G1 and G2. Using the same analytical protocols as in the case of mission 1, no detectable differences could be observed either in UV or in TICs (total ion chromatograms). The overlaid HPLC chromatograms of S1, G1, S2, G2 and acetonitrile were mainly superimposed (Fig. S6). There were only two intense peaks at Rt 5.5 and 6.38 min, which were dominant in the G2 sample. A detailed study revealed that the masses of these ions (359.150 Da and 387.181 Da—Fig. S6) are also present in the acetonitrile sample with high intensities and in all the other samples with low intensities. Consequently, these ions cannot be considered RDV related. All the other peaks in the chromatograms showed very similar shapes and intensities. Significant differences could not be observed. Some characteristic RDV degradation products were present in each sample. In fact, two detectable degradation products were identified at RDV Deg3 (Rt 4.19 min with m/z 527.202 Da) and RDV Deg4 (Rt 4.66 min with m/z 621.243 Da together with its isotope 4.78 (622.228). Interestingly, the “acidic” degradation products (RDV Deg1 and Deg2—according to the stress stability study referenced) were completely missing in these samples.

In summary, in the second mission the RDV sample analysis obtained on Earth and in space has comparable results. We observed only limited degradation, which seems to be promising, particularly in terms of long-term space missions of cyclodextrin-stabilized drug complexes. It was suggested that radiation, microgravity, and the harsh conditions related to transiting did not have any impact on the stability of the cyclodextrin complex.

### Controlling the parameters of the different steps in the experimentation

The difference in stability results obtained on Earth and in space in the first and the second missions have raised an important question. What is the main reason for not being successful in reproducing the stability results of the first mission?

We think the complexity of our experiments revealed several parameters that are difficult to control and precisely monitor during the entire journey. For example, the hardly controllable conditions during shuttle launch and transition period of the sample might play an important role in the stability. As discussed in a recent paper^35^, samples returning to Earth suffer from unavoidable transit times (~ 45 h), passing through the Earth’s atmosphere and then the intercontinental travel, during which they encounter a range of conditions including radiation, temperature changes, temporary high gravity exposure, magnetic field and vibration that are typically inadequately or even not at all monitored. It should be noted that for comparability reasons the transition and storage condition of the terrestrial control sample should be kept under similarly strict control.

Table 3 lists the different stages of the sample’s journey together with the corresponding events happened with the samples. We deemed noteworthy to introduce 3 categories for our ability to control the experimental conditions of our samples: A = directly controllable, B = indirectly controllable, C = not controllable activities.

**Table 3:** Different stages and activities involved in the sample’s journey.

Steps	Event	Location	Duration (day)	Controllability
1	Preparation of samples	Budapest	1	A
2	Transfer of samples to JAMSS/CFS	Budapest to Tokyo	4	C
3	Storage of samples at CFS	Tokyo	17	B
4	Preparation of samples for launch	Tokyo	1	B
5	Sending samples to SpaceX launch site/to Florida	Tokyo to FL/USA	6	C
6	Storage of samples in Florida/ KIRARA Cube loading	FL/USA	6	C
7	Rocket launch/ SpaceX Cargo flight to ISS	FL/USA to ISS on orbit	0.5	C
8	KIRARA cube experiment at ISS	ISS on orbit	31	A
9	Returning samples to Earth	ISS to FL/USA	1	C
10	Storage of samples in Florida	FL/USA	n.a	C
11	Return of samples to JAMSS/CFS	FL/USA to- Tokyo	8 + 3 = 11	C
12	Storage of samples at CFS	Tokyo	4	B
13	Transfer of samples to Budapest	Tokyo to Budapest	7	C
14	Storage of samples in Budapest	Budapest	Indefinite	A
15	Analytical tests	Budapest	Indefinite	A
16	Further storage	Budapest	Indefinite	A

The terrestrial control samples followed the same path except steps 7, 8, 9. The A, B, C categories are described above.

Statistically, approximately one third of the processes are directly controllable; another one fifth are indirectly controllable, and nearly half of the processes are not controllable at all. We assume that the high proportion of the uncontrollable processes has a major impact on the outcome of the experiments.

**Fig. 3:**
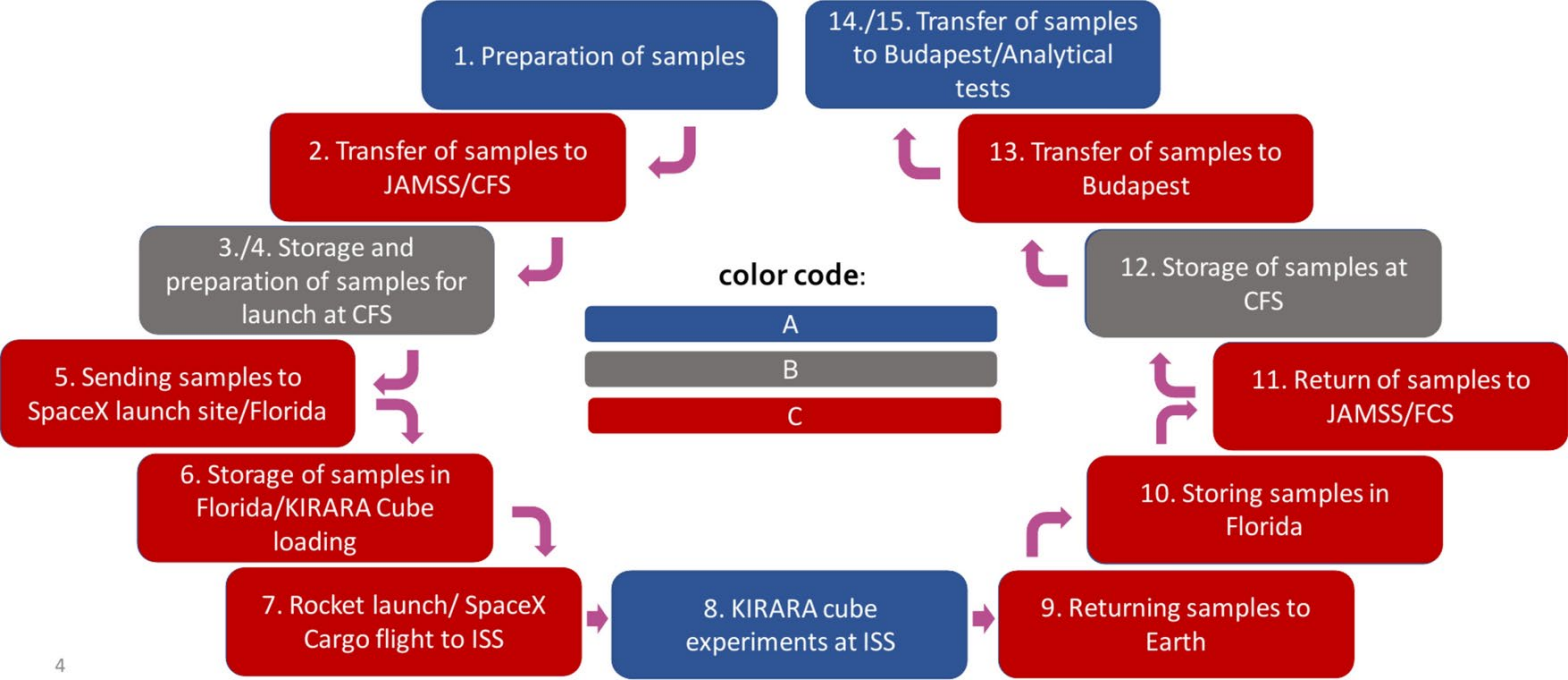
Representation of the entire journey according to the various degree of controllability. A, B, C categories correspond to Table 3.

The limited controllability (Fig. 3) also highlighted the problems of standardization, including strict parameter monitoring and control. Presently, no standard and authorized procedures are available for drug stability testing in space as well as for their counterparts remaining on Earth. It is obvious, that the terrestrial processes are not suitable and adaptable for the space environment, thus, new ones are needed. Consequently, it is strongly recommended to collect and study all the processes and the existing standards in detail before starting highly complex research experiments.

On the other hand, the purpose, the approach, and the drive of the standards can be quite different. Different set of standards are developed to unrelated fields, e.g. for airway transportation regarding temperature, magnetic or radiation protection, while different standards are applied for rocket launches to the ISS. Efforts should be made to integrate the standards to each and every step of the entire journey including the parallel events of the terrestrial counter-samples.

The above outlined picture makes it clear that neither Earth vs. space processes, nor a combination of both can be adequately controlled unless the fundamental issues of standardization are addressed within the context of the whole journey. Intuitively, it is obvious to us that some of the reproduction problems we have experienced may be related to inadequate solutions of the missing standardization issues.

We hope that the growing interest in similar complex on-orbit studies related to space chemistry will trigger a greater focus on the standardization issues discussed above.

## Conclusion

Space chemistry^36,37^ under microgravity opens unique opportunities to develop new and more efficient drugs used both in space and on Earth. Microgravity could produce unique drug forms, including crystals that grow larger with more regular morphology and provide more stable proteins or other chemical entities of therapeutic relevance^38^. In the absence of a preferred direction of gravity, drug formulation and crystal formation can occur in a convection and sediment-free manner. On the other hand, many drugs have a limited lifetime in space due to increased degradation^39^ and stability difference on Earth and in space of other API’s has already been demonstrated earlier^39^. In the meantime, we were unable to find examples in the literature for reproduction of drug discovery or even chemical experiments in space. This is somehow in contrary to the generally accepted principle, which requires repetition of the chemical experimentations. Thus, after the unexpected, encouraging result, it was mandatory to attempt to reproduce these findings.

In the presented two space experiments, we could draw the following conclusions:

- The cyclodextrin derivative applied in the experiments (SBECD) remained fully stable during both experiments on orbit. This is a significant outcome since the stability of related drug complexes including cyclodextrin derivatives in space has not been discussed in the literature up to now.
- The stability and solubility of the dissolvable powder formulation of remdesivir has not decreased, which confirmed the protective effect of the CD complex in space as well.
- Microgravity has not improved the solubility of RDV complex as well as did not change its stoichiometry.

On the other hand, the above observations also confirmed the particular need of standardized protocols for further pharmaceutical studies in space including the comparative trials on Earth that can ensure the reproducibility of the results. The high complexity and limited controllability require strict parameter monitoring and control including the following steps and activities:

- Transfer of samples to and from the research and packing locations to the rocket launch sites
- Storage of the samples at any transitional and final destination on Earth
- Rocket launch to space and return to Earth
- Storage of the samples at the space station

In particular, the last two steps contain several not fully monitored or hardly controllable parameters and factors such as different forms of radiation, vacuum, magnetic field, temperature changes, as well as excessive vibration and gravity fluctuation during shuttle launch and transition. Based on the above variable conditions it is strongly recommended to collect and study all these processes and then propose appropriate monitoring and standardization as much as possible.

The final conclusion of the present repetitive study is that since all the above factors could significantly influence the efficacy and chemical stability of the drug components the stability of drugs used for space missions cannot be evaluated by drug stability guidelines based on terrestrial environmental factors and new, adaptive, multi-level, standard procedures are needed.

## Supplementary Information

Supplementary Information.

## Data Availability

The datasets used and/or analyzed during the current study available from the corresponding author on reasonable request.
