# Supplementary material for: Repetitive stability study of remdesivir/cyclodextrin complex on the international space station
Source: Sci Rep. 2025 Feb 4;15:4182. doi: 10.1038/s41598-024-81428-5 (PMC11794431; doi:10.1038/s41598-024-81428-5)
Supplement: Supplementary file 1 — Supplementary Information. [file 41598_2024_81428_MOESM1_ESM.docx]

**Extending anti-COVID drug discovery to Space: Repetitive stability study of Remdesivir/CD complex on ISS**

**(Supplementary Material)**

György Dormán^1#^, Balázs Buchholcz^1#^, István Puskás^2^, Pál Szabó^3^, Erzsébet Varga^2^, Lajos Szente^2^, György M. Keserű^3^ and Ferenc Darvas^1^*

^1^Innostudio, Inc., Záhony u. 7., Budapest, H-1031, Hungary

^2^Cyclolab Cyclodextrin Research & Development Laboratory Ltd, Illatos út 7., Budapest, H-1097, Hungary

^3^Research Centre for Natural Science and National Drug Research and Development Laboratory, Magyar Tudósok körútja 2., Budapest, H-1117, Hungary

^#^ G. D. and B. B. contributed equally to this work

Corresponding author: Dr. Ferenc Darvas, ferenc.darvas@innostudio.org, df.private@gmail.com

Content

[1. Reproducibility of the HPLC method for RDV purity and chemical degradation analysis (Method B) 1](#_Toc178797250)

[2. HPLC degradation results 2](#_Toc178797251)

[2.1. SBECD degradation comparison G1/S1 samples (fingerprint investigation) (1st mission): 2](#_Toc178797252)

[2.2. RDV degradation analysis of the G1/S1 neutral samples delivered from the 1^st^ mission 3](#_Toc178797253)

[2.3. RDV degradation analysis of the G1/S1 neutral samples delivered from the 2nd mission 5](#_Toc178797254)

[3. RDV degradation products’ identification 5](#_Toc178797255)

# Reproducibility of the HPLC method for RDV purity and chemical degradation analysis (Method B)

| UV | | | | | | | |
| --- | --- | --- | --- | --- | --- | --- | --- |
|  | Run 1 |  | Run 2 |  | UV |  | UV |
| Sample ID | Sample1 | Sample2 | Sample1 | Sample2 | Run1 | Run2 | SD overall |
| 0123 | 3.30E+05 | 3.29E+05 | 3.61E+05 | 3.56E+05 | 0.107251 | 0.947811 | 4.810862 |
| 0125 | 3.55E+05 | 3.51E+05 | 3.03E+05 | 3.01E+05 | 0.760545 | 0.51477 | 9.018156 |
| 0126 | 3.67E+05 | 3.64E+05 | 3.41E+05 | 3.38E+05 | 0.657594 | 0.812407 | 4.324446 |
| 0130 | 3.64E+05 | 3.61E+05 | 3.96E+05 | 3.93E+05 | 0.721836 | 0.519997 | 4.893552 |
| 0201 | 3.92E+05 | 3.91E+05 | 3.85E+05 | 3.80E+05 | 0.252925 | 0.868523 | 1.404063 |
| MS | | | | | | | |
|  | Run 1 |  | Run 2 |  | MS |  | MS |
| Sample ID | Sample1 | Sample2 | Sample1 | Sample2 | Run1 | Run2 | SD overall |
| 0123 | 5.43E+07 | 6.07E+07 | 4.93E+07 | 5.89E+07 | 7.933963 | 12.5095 | 9.144691 |
| 0125 | 5.92E+07 | 5.09E+07 | 5.45E+07 | 5.23E+07 | 10.59986 | 2.860207 | 6.653339 |
| 0126 | 6.01E+07 | 5.79E+07 | 5.70E+07 | 5.80E+07 | 2.624017 | 1.217136 | 2.24612 |
| 0130 | 6.12E+07 | 6.46E+07 | 5.76E+07 | 6.71E+07 | 3.855618 | 10.73727 | 6.583686 |
| 0201 | 6.55E+07 | 6.45E+07 | 6,41E+07 | 6.31E+07 | 1.055627 | 1.045424 | 1.518562 |

Table S1. Reproducibility of the RDV HPLC UV/MS measurements

# HPLC degradation results

## SBECD degradation comparison G1/S1 samples (fingerprint investigation) (1st mission):

Figure S1. HPLC chromatogram of samples G1 (blue) and S1 (red) by SBECD fingerprint method (Method A)


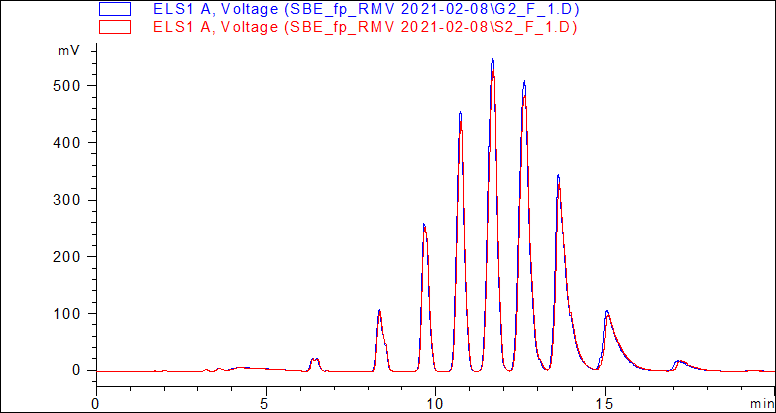


Figure S2. HPLC chromatogram of samples G2 (blue) and S2 (red) by SBECD fingerprint method (Method A)

No SBECD degradation product was detected in any of the samples.

## RDV degradation analysis of the G1/S1 neutral samples delivered from the 1^st^ mission

A series of major and minor peaks were obtained in both the UV (Fig. S3) and total ion chromatograms (TICs). The overlaid chromatograms of G1 (blue), S1 (pink) and acetonitrile as a control (orange) help visualize the differences in the samples. Peaks present only in blue and/or pink but not in orange are compounds related to RDV. Peaks present only in the real samples but not in the acetonitrile control have been discussed. (Fig. 2 and Fig. S4).

Figure S3. HPLC trace of G1 and S1 samples (UV detection)


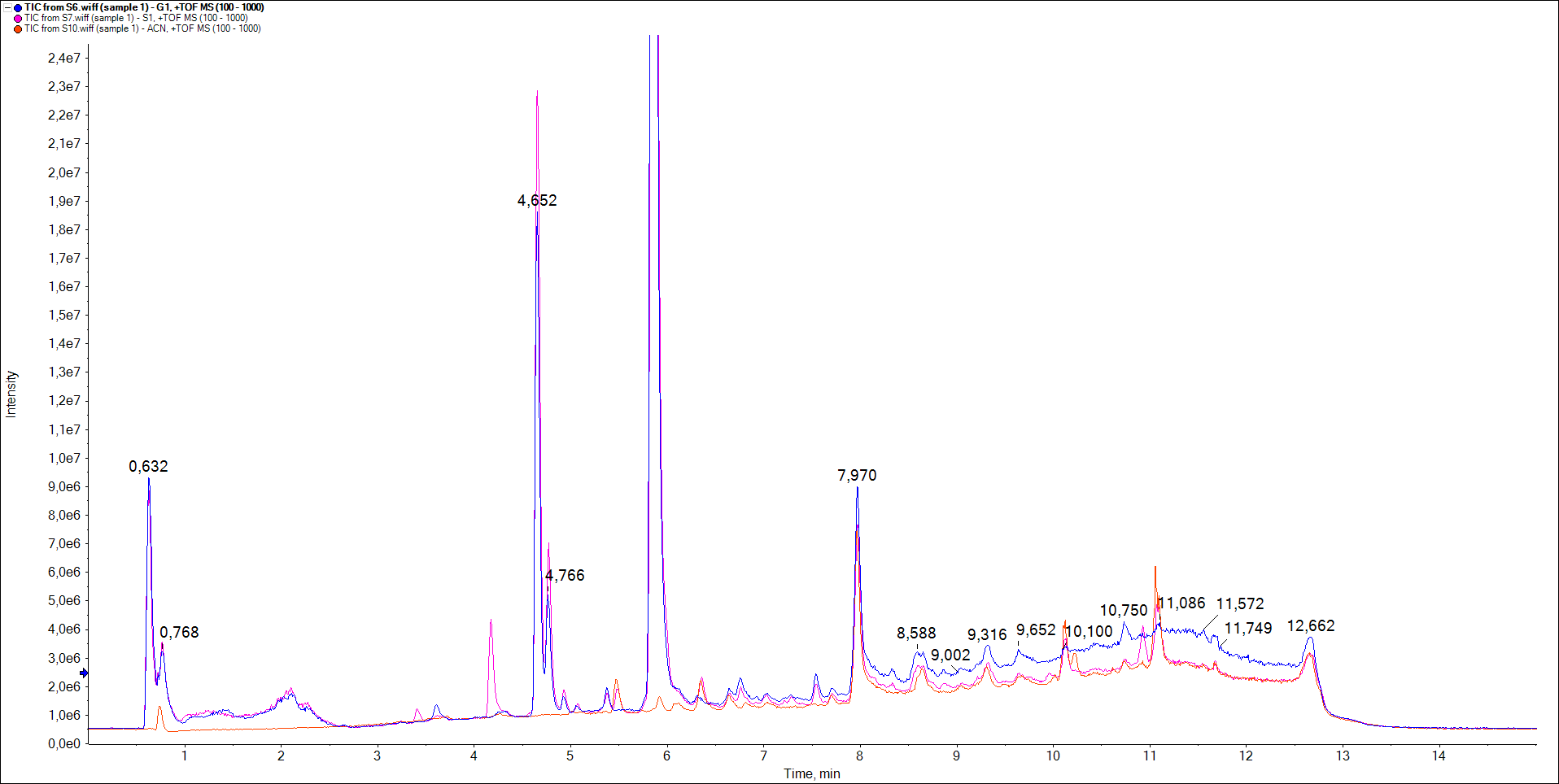


Figure S4. Total-ion chromatogram (TIC) of G1 (blue), S1 (pink) and acetonitrile blank (orange) of G1 and S1 samples (TIC)


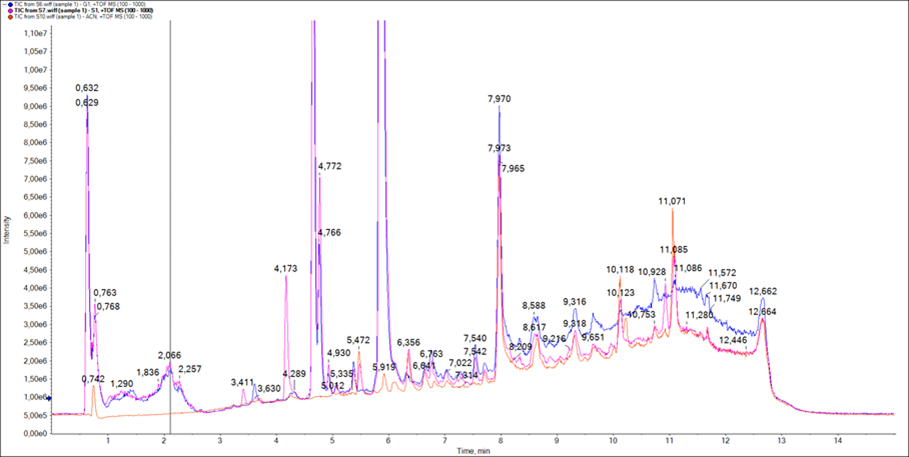


Figure S5. Zoomed total-ion chromatogram (TIC) of G1 (blue), S1 (pink) and acetonitrile blank (orange)

## RDV degradation analysis of the G1/S1 neutral samples delivered from the 2nd mission

Figure S6. Zoomed total-ion chromatogram (TIC) of G1 (blue), S1 (pink) and acetonitrile blank (orange). Acetone peaks at Rt = 5.5 and 6.38 mins. (2nd mission)

# RDV degradation products’ identification

Fig. S7A. Degradation products formed under acidic stress conditions (based on Ref.^[[1]](#endnote-1)^)

Fig. S7B. Degradation products formed under basic stress conditions (based on Dadinaboyina, et al. New J. Chem. 2021, 45, 7217-7224.)

Fig. S7C. Degradation products formed under neutral (DP5) and oxidative (DP9) stress conditions (based on Dadinaboyina, et al. New J. Chem. 2021, 45, 7217-7224.)


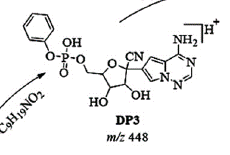


Figure S8. Degradation product at 2.657 min; (M+H=448) (RDV Deg1)


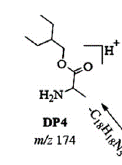


Figure S9. Degradation product at 3.6 min; (M+H=174) (RDV Deg2)


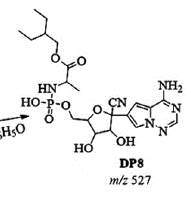


Figure S10. Degradation product at 4.173 min; M+H=527 (RDV Deg3)


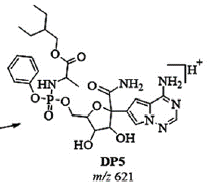


Figure S11. Degradation product at 4.652 min; (M+H=621) (RDV Deg4)

1. **References**

   () Dadinaboyina, S. B.; Yerra, N. V.; Adimoolam, B. M.; Parsa, S.; Bathini, N. B.; Thota, J. R. Identification and characterization of degradation products of Remdesivir using liquid chromatography/mass spectrometry. *New J. Chem.* **2021**, *45*, 7217-7224. [↑](#endnote-ref-1)
